# On the merits and potential of advanced neuroimaging techniques in COVID-19: A scoping review

**DOI:** 10.1016/j.nicl.2024.103589

**Published:** 2024-03-06

**Authors:** Noa van der Knaap, Marcel J.H. Ariës, Iwan C.C. van der Horst, Jacobus F.A. Jansen

**Affiliations:** a Department of Intensive Care Medicine, Maastricht University Medical Center, Maastricht, the Netherlands; b Department of Radiology & Nuclear Medicine, Maastricht University Medical Center, Maastricht, the Netherlands; c Research Institute of Mental Health & Neuroscience, Maastricht University, Maastricht, the Netherlands; d Cardiovascular Research Institute Maastricht (CARIM), Maastricht University, Maastricht, the Netherlands; e Department of Electrical Engineering, Eindhoven University of Technology, Eindhoven, the Netherlands

**Keywords:** COVID-19, long-COVID, Advanced neuroimaging, Magnetic resonance imaging, Positron emission tomography

## Abstract

Many Coronavirus Disease 2019 (COVID-19) patients are suffering from long-term neuropsychological sequelae. These patients may benefit from a better understanding of the underlying neuropathophysiological mechanisms and identification of potential biomarkers and treatment targets. Structural clinical neuroimaging techniques have limited ability to visualize subtle cerebral abnormalities and to investigate brain function. This scoping review assesses the merits and potential of advanced neuroimaging techniques in COVID-19 using literature including advanced neuroimaging or postmortem analyses in adult COVID-19 patients published from the start of the pandemic until December 2023. Findings were summarized according to distinct categories of reported cerebral abnormalities revealed by different imaging techniques. Although no unified COVID-19-specific pattern could be subtracted, a broad range of cerebral abnormalities were revealed by advanced neuroimaging (likely attributable to hypoxic, vascular, and inflammatory pathology), even in absence of structural clinical imaging findings. These abnormalities are validated by postmortem examinations. This scoping review emphasizes the added value of advanced neuroimaging compared to structural clinical imaging and highlights implications for brain functioning and long-term consequences in COVID-19.

## Highlights

• Advanced neuroimaging complements structural clinical imaging findings in COVID-19.
• Advanced neuroimaging findings reflect hypoxic, vascular, and inflammatory damage.
• In vivo advanced neuroimaging findings are supported by postmortem histology.
• Cerebral abnormalities are likely attributed to indirect viral infection.
• Understanding COVID-19 neuropathology demands multiparametric imaging protocols.

## Graphical abstract

**Figure:**
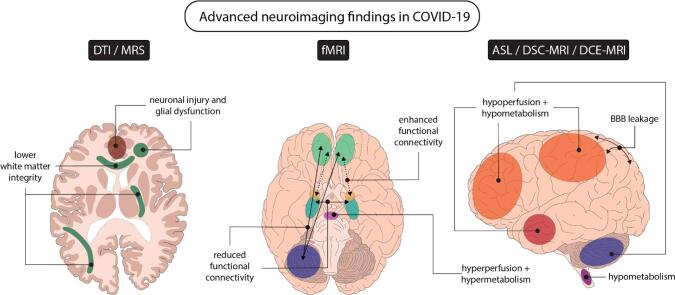

## 1 Introduction

Since the end of 2019, a novel coronavirus – severe acute respiratory syndrome coronavirus 2 (SARS-CoV-2) – has had a global impact on society and healthcare systems. This highly contagious virus has resulted in the Coronavirus Disease 2019 (COVID-19) pandemic, with a continuously increasing number of over 650 million confirmed cases of infection and close to seven million deaths worldwide (World Health Organization Coronavirus (COVID-19) Dashboard, 2023). Although the pulmonary system is primarily affected, there is growing evidence of cerebral involvement in COVID-19 (Newcombe et al., 2021).

Multiple pathways of viral neuroinvasion have been proposed, including transsynaptic transfer across infected neurons, intracranial entry via the olfactory nerve, by infection of the vascular endothelium, or through viral-infected leukocyte migration across a permeable blood–brain barrier (Bauer et al., 2022, Lima et al., 2020). Cerebral damage may also be a consequence of associated systemic illness, such as prolonged hypoxia, systemic inflammation, and hypercoagulation (Crook et al., 2023). Common neurological complaints in both mildly and severely affected COVID-19 patients are anosmia and ageusia (Sudre et al., 2021), suggesting involvement of the olfactory system. In early 2020, neuroradiological case reports revealed cerebral abnormalities in acute hospitalized COVID-19 patients (Moriguchi et al., 2020). Since then, clinical neuroimaging protocols have been extensively utilized, revealing a wide range of structural cerebral abnormalities in the acute stage of the disease (Egbert et al., 2020, Kim et al., 2021) and in patients with post-acute sequelae of COVID-19 infection (so-called ‘long-COVID syndrome’) (Vasilev et al., 2023).

The reported structural imaging findings include signal abnormality of the olfactory bulb, parenchymal diffusion abnormalities, white matter hyperintensities, encephalitis, microbleeds, stroke, and thickening and enhancement of arterial vessel walls. Findings from neuroimaging studies have been complemented with postmortem brain studies in COVID-19 patients, which have revealed microvascular cerebral injury (Lee et al., 2021), microglial activation and extensive neuroinflammation (Matschke et al., 2020, Schurink et al., 2020), thrombi, and neutrophilic plugs (Schurink et al., 2020).

Most imaging studies in COVID-19 have used structural clinical imaging protocols readily available on clinical scanners. Although indispensable, standard clinical imaging protocols merely reveal the tip of the iceberg, particularly regarding long-term sequelae (Vasilev et al., 2023). These protocols are restricted to visualization and detection of static abnormalities of macroscale anatomy. Static changes may only occur at later stages or be an end product of pathological processes, whereas functional or preceding microstructural abnormalities remain undetected. Advanced neuroimaging acquisition and analysis techniques – such as MRI diffusion tensor imaging (DTI), MRI perfusion-weighted imaging (PWI), functional magnetic resonance imaging (fMRI), and radiotracer positron emission tomography (PET) – allow imaging beyond the macroscale anatomy together with opportunities to study brain functioning (van Bussel et al., 2017).

This scoping review aims to provide an overview of the merits and potential of advanced neuroimaging techniques in patients with a proven COVID-19 infection. The review focuses on three questions:

1) What advanced imaging techniques have been used so far?
2) What additional information is obtained from studies employing advanced imaging techniques?
3) Do these insights align with findings from (a) structural clinical imaging protocols and (b) postmortem examinations?

## 2 Methods

**Fig. 1:**
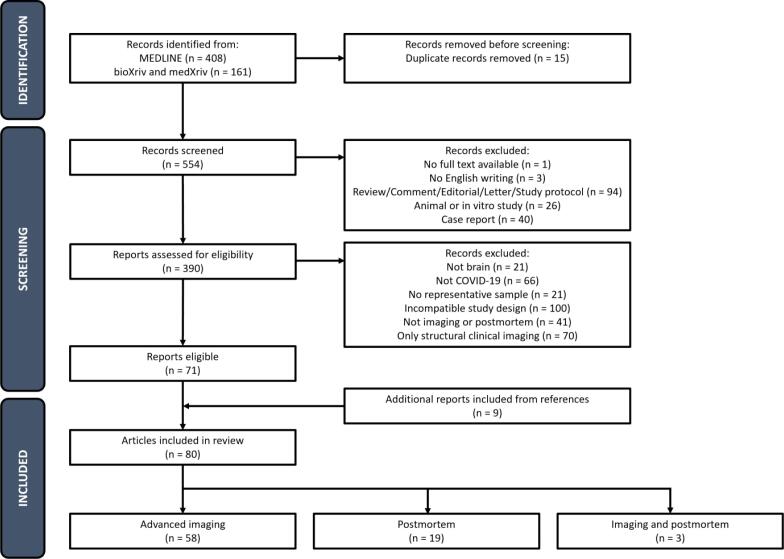
PRISMA flowchart of the article selection process (16).

The scoping review method was selected with the aim to summarize and map current advanced neuroimaging findings in COVID-19 and to identify research gaps in the existing literature to facilitate future research (Tricco et al., 2018). We performed a literature search using the MEDLINE, bioRxiv, and medRxiv databases. These databases were searched for articles using the following MeSH terms: 'COVID-19′ or 'SARS-CoV-2′; 'brain'; and 'magnetic resonance imaging' or 'computed tomography' or 'positron emission tomography' or 'single-photon emission computed tomography' or 'postmortem' or 'autopsy' (see Supplementary Table 1 for details). For general criteria, all articles needed to concern adult patients with confirmed infection of the Sars-CoV-2 virus (regardless of hospitalization status, virus variant, disease stage, and disease severity) and include advanced imaging or postmortem pathological assessment of the brain. In this scoping review, we specifically focused on advanced neuroimaging techniques and eliminated articles that only reported findings from structural clinical imaging protocols. Structural clinical imaging protocols typically include structural MRI sequences (i.e., T_1_-weighted, T_2_-weighted, fluid attenuation inversed recovery (FLAIR), diffusion weighted imaging (DWI), and susceptibility weighted imaging (SWI)) and/or CT imaging. Postmortem studies were included to provide histological support for COVID-19 findings. The literature search was last updated on December 6th, 2023. Retrieved articles were manually screened for eligibility based on title and abstract. Full-text articles were evaluated for final selection. References from included articles were screened for supplemental article inclusion. Case reports and case series (n < 10 patients), reviews, animal studies, studies published before 2019, and reports not written in English were excluded. The study selection process is presented in Fig. 1.

## 3 Results

The database search resulted in 554 research articles. After the screening procedure, eighty articles were included in this review. Fifty-eight articles used one or more advanced neuroimaging techniques in addition to structural clinical imaging. Articles that solely reported structural clinical imaging (n = 70) were excluded from further discussion in this review (but see other reviews on this topic (Parsons et al., 2021, Moonis et al., 2021, Ladopoulos et al., 2021). Three postmortem studies reported on neuroimaging findings and another nineteen studies reported on postmortem histological assessment only.

**Fig. 2:**
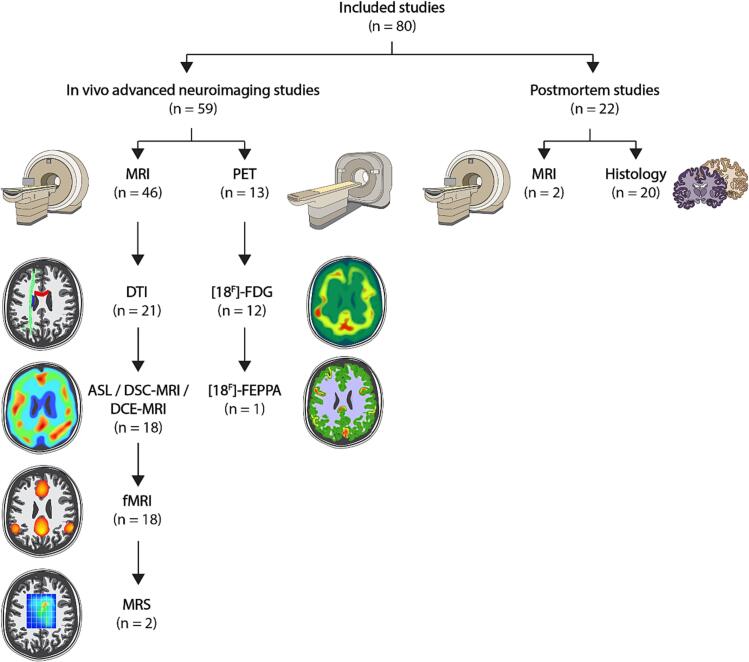
**Overview of advanced neuroimaging techniques and postmortem analyses in COVID-19.***MRI = magnetic resonance imaging; DTI = diffusion tensor imaging; fMRI = functional magnetic resonance imaging; ASL = arterial spin labelling; DSC-MRI = dynamic susceptibility contrast magnetic resonance imaging; DCE-MRI = dynamic contrast-enhanced magnetic resonance imaging; MRS = magnetic resonance spectroscopy; PET = positron emission tomography; [18^F^]-FDG = fluorodeoxyglucose; [18^F^]-FEPPA = [18^F^]-N-(2-(2-Fluoroethoxy)benzyl)-N-(4-phenoxypyridin-3-yl).* *One study used advanced neuroimaging for in vivo assessment and histology of postmortem material (different subjects) and is therefore included in both categories. Hence, the total number of 80 included studies is split into 59 in vivo advanced neuroimaging studies and 22 postmortem studies.

Interestingly, MRI was the most frequently used neuroimaging modality (78 %), followed by PET (22 %). Both modalities can be utilized for different purposes depending on the MRI sequences or PET radiotracer used (Fig. 2). The MRI techniques used were diffusion tensor imaging (DTI) (46 %), arterial spin labelling (ASL) or dynamic susceptibility contrast MRI (DSC-MRI) or dynamic contrast-enhanced MRI (DCE-MRI) (39 %), functional MRI (39 %), and magnetic resonance spectroscopy (MRS) (4 %). Multiparametric MRI was applied in ten studies. Most PET studies used fluorodeoxyglucose ([18^F^]-FDG) as a radiotracer (92 %) and one study used [18^F^]-N-(2-(2-Fluoroethoxy)benzyl)-N-(4-phenoxypyridin-3-yl) ([18^F^]-FEPPA) (8 %).

**Fig. 3:**
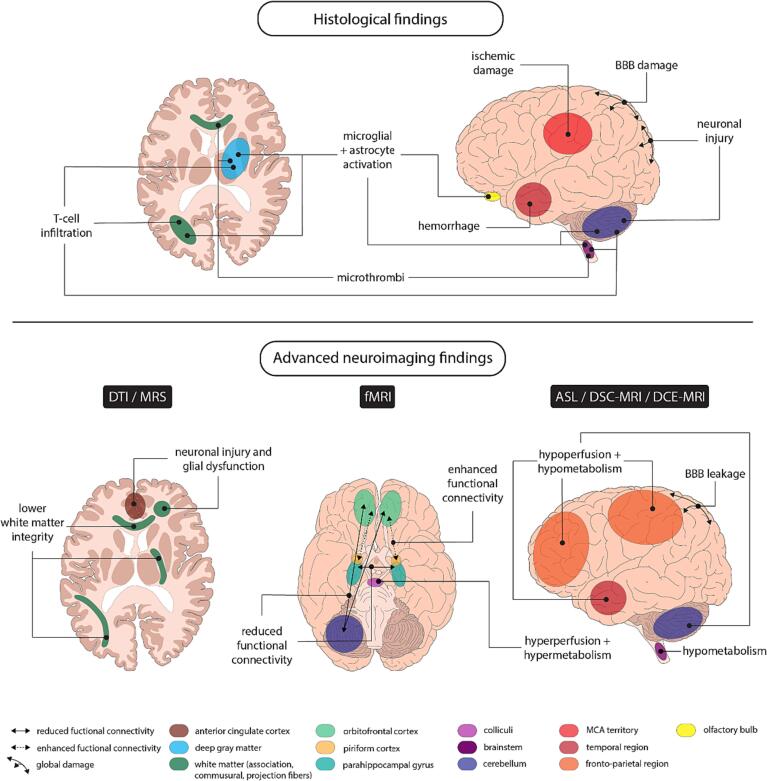
**Graphical summary of histological and advanced neuroimaging findings.** The most common abnormalities in COVID-19 patients relative to healthy control subjects are depicted.

The results of the included articles are mapped (Fig. 3) and summarized below according to the different types of distinguished cerebral abnormalities. Of the articles that used advanced neuroimaging techniques, 47 % included only hospitalized patients in their study sample, 27 % included only non-hospitalized patients, and 25 % included both hospitalized and non-hospitalized patients (Table 1). Most of the included articles excluded patients with pre-existing neurological or neuropsychiatric disorders (69 %). While analyzing these results, we considered the studied patient samples (e.g., COVID-19 patients with or without long-COVID syndrome), the timing of the moment of brain imaging relative to the infection period (e.g., pre-infection versus months or years post-infection), the COVID-19 patient recruitment period (i.e., as a proxy for the COVID-19 pandemic period and dominant virus variants), and if neuroimaging findings in COVID-19 were compared to other patient groups or healthy controls (see Table 1). Additionally, Table 1 provides an overview of whether clinical symptoms (objective and/or subjective) at the time of neuroimaging were recorded and if a relation to any neuroimaging markers. Finally, results from postmortem studies are summarized and discussed considering the earlier presented imaging findings (Fig. 3).

**Table 1:** Advanced imaging study design and main findings.

Article	Imaging modality	Approximate imaging timeline COVID-19 sample^a^	COVID-19 subject recruitment period	COVID-19 hospitalization status	Pre-existent neurological disease	COVID-19 sample	Control sample	Main imaging findings	Subjective / Objective neuropsychological complaint measurements^b^	Relation neuropsychological measures to imaging
DTI										
Qin et al. 2021 (42)	3 T MRI	3 m post-infection	March 2020	Hospitalized	Excluded	51 COVID-19	31 healthy	White matter alterations: lower FA, tract volume, and tract length	None	–
Douaud et al. 2022 (26)	3 T MRI	T1: pre-infection T2: 4.5 m post-infection	Start pandemic – February 2021	Hospitalized and non-hospitalized	Excluded	401 COVID-19	384 healthy	White matter alterations: greater longitudinal increase in MD in orbitofrontal cortex	Objective	Not reported
Scardua Silva et al. 2021 (57)	3 T MRI	T1: 1.5 m post-infection	Not reported	Non-hospitalized	Not reported	87 COVID-19	55 healthy	No changes in diffusion	Subjective + objective	Correlation between white matter microstructural abnormalities and cognition (attention and cognitive flexibility)
Yang et al. 2021 (58)	3 T MRI	T1: 3 m post-infection	April 2020 - June 2020	Hospitalized	Excluded	28 COVID-19	27 healthy	White matter alterations: reduced FA and increased MD and RD	Subjective	Correlation between white matter microstructural abnormalities and mental health scores.
Fischer et al. 2022 (54)	3 T MRI	T1: acute stage of infection	July 2020 - March 2021	Hospitalized	Excluded	12 COVID-19 with disorder of consciousness	14 healthy + 18 traumatic brain injury	White matter alterations: reduced FA (similar in TBI group)	None	–
Huang et al. 2022 (59)	3 T MRI	T1: 12 m post-infection	February 2020 - April 2020	Hospitalized	Excluded	22 COVID-19	21 healthy	White matter alterations: reduced FA and lower fraction of dendrites and axons	Objective	Not reported
Esposito et al. 2022 (27)	3 T MRI	T1: 0.5 m post-infection	April 2020 - December 2020	Hospitalized and non-hospitalized	Excluded	27 COVID-19 with hyposmia	18 healthy	Structural connectivity: increased connectivity within olfactory network	Objective	No correlation between structural connectivity and olfaction
Tian et al. 2022 (43)	3 T MRI	T1: 3.5 m post-infection T2: 10 m post-infection	March 2020	Hospitalized	Excluded	34 COVID-19	31 healthy	White matter alterations: lower tract volume compared to controls; within covid group over time increase in tract volume	Subjective	Not reported
Petersen et al. 2022 (60)	3 T MRI	T1: 9.5 m post-infection	March 2020 - December 2020	Hospitalized and non-hospitalized	Not reported	223 COVID-19	223 healthy	White matter alterations: higher MD and extracellular free-water fraction	Subjective + objective	Correlation between white matter microstructural abnormalities and cognition (executive function, working memory, and verbal fluency) in the COVID-19 group
Carvalho Bispo et al. 2022 (53)	3 T MRI	T1: 3 m post-infection	October 2020 - May 2021	Non-hospitalized	Excluded	56 COVID-19	37 healthy	White matter alterations: lower fiber density	Subjective + objective	Correlation between white matter microstructural abnormalities and fatigue and cognition (processing speed and visual memory)
Teller et al. 2022 (62)	3 T MRI	T1: initial visit T2: 3 m after initial visit	May 2020 - September 2021	Non-hospitalized	Excluded	39 COVID-19	14 healthy	White matter alterations: reduced and increased diffusion restriction	None	–
Díez-Cirarda et al. 2022 (63)	3 T MRI	T1: 11 m post-infection	November 2020 - December 2021	Hospitalized and non-hospitalized	Excluded	86 long-COVID	36 healthy	White matter alterations: lower MD and AD	Subjective + objective	No correlation with white matter microstructural abnormalities and cognition
Planchuelo-Gómez et al. 2023 (56)	3 T MRI	T1: 10 m post-infection	March 2020 - April 2020	Hospitalized and non-hospitalized	Excluded	40 COVID-19 with persistent headache	41 healthy	White matter alterations: lower FA and higher RD	None	–
Campabadal et al. 2023 (25)	3 T MRI	T1: 11 m post-infection	April 2021 - November 2021	Hospitalized and non-hospitalized	Excluded	23 COVID-19 with olfactory dysfunction	25 COVID-19 without olfactory dysfunction	White matter alterations: higher MD and RD	Objective	Correlation between white matter microstructural abnormalities and olfaction
Huang et al. 2023 (61)	3 T MRI	T1: 12 m post-infection T2: 26.5 m post-infection	February 2020 - April 2020	Hospitalized	Excluded	17 COVID-19	13 healthy	White matter alterations: higher FW compared to controls. Higher RD and lower ODI, V_ic_, V_iso_ in T2 versus T1	Objective	Correlation between white matter microstructural abnormalities and cognition (memory)
Paolini et al. 2023 (55)	3 T MRI	T1: 6 m post-infection	January 2021 - January 2022	Hospitalized	Excluded	29 long-COVID	29 COVID-19 without long-COVID	White matter alterations: higher MD and AD	Subjective	White matter microstructural abnormalities in COVID-19 patients with persisting cognitive complaints compared to patients without persistent complaints
Tassignon et al. 2023 (66)	3 T MRI	T1: 1–2 m post-infection T2: 3–4 m post-infection	Not reported	Hospitalized	Not reported	12 COVID-19	None	Structural connectivity: decrease characteristic path length	Objective	No correlation between structural connectivity and cognition
Carvalho Bispo et al. 2023 (28)	3 T MRI	T1: 3 m post-infection	October 2020 - May 2021	Non-hospitalized	Excluded	38 COVID-19	24 healthy	Structural connectivity: reduced integration and increased segregation within the olfactory system	Objective	Correlation between structural connectivity and olfaction
Zhang et al. 2023 (65)	3 T MRI	T1: pre-infection T2: 4 m post-infection	Start pandemic - March 2023	Hospitalized	Excluded	224 COVID-19	192 healthy	No change in diffusion restriction after infection	None	–
Díez-Cirarda et al. 2023 (37)	3 T MRI	T1: 11 m post-infection	November 2020 - December 2021	Hospitalized and non-hospitalized	Excluded	84 long-COVID	33 healthy	Higher intracellular volume fraction and higher ODI in hippocampus	Subjective + objective	Correlation between hippocampal microstructural abnormalities and cognition (attention, memory, and processing speed)
Lathouwers et al. 2023 (64)	3 T MRI	T1: post-infection (unspecified)	Not reported	Hospitalized	Not reported	20 COVID-19	18 healthy	White matter alterations: lower whole-brain FC and lower tract-specific FD and FDC	Objective	Correlation between white matter microstructural changes and cognition (motor speed, attention, and executive function)
ASL, DSC-MRI, DCE-MRI										
Chougar et al. 2020 (38)	3 T MRI	T1: 0.5–1 m post-infection	March 2020 - May 2020	Hospitalized	Excluded	73 COVID-19	None	Hypoperfusion	None	–
Klironomos et al. 2020 (45)	1.5 T MRI 3 T MRI	T1: 1 m post-infection	March 2020 - May 2020	Hospitalized	Included	174 COVID-19	None	No perfusion abnormalities	None	–
Henry-Feugeas et al. 2020 (39)	1.5 T MRI	T1: acute stage of infection	March 2020 - April 2020	Hospitalized	Not reported	25 COVID-19 with neurological symptoms	None	Hypoperfusion and hyperperfusion	None	–
Qin et al. 2021 (42)	3 T MRI	3 m post-infection	March 2020	Hospitalized	Excluded	51 COVID-19	31 healthy	Hypoperfusion	None	–
Hosp et al. 2021 (46)	3 T MRI PET	T1: 1 m post-infection	June 2020 - January 2021	Hospitalized	Excluded	13 COVID-19	30 healthy	No perfusion abnormalities	Subjective + objective	Not reported
Chammas et al. 2021 (44)	3 T MRI	T1: acute stage of infection T2: 1 m post-infection	March 2020 - August 2020	Hospitalized	Not reported	112 COVID-19	25 healthy	Hyperperfusion	None	–
Lambrecq et al. 2021 (40)	3 T MRI	T1: acute stage of infection	March 2020 - June 2020	Hospitalized	Not reported	78 COVID-19	None	Hypoperfusion	None	–
Tian et al. 2022 (43)	3 T MRI	T1: 3.5 m post-infection T2: 10 m post-infection	March 2020	Hospitalized	Excluded	34 COVID-19	31 healthy	Hypoperfusion	Subjective	Not reported
Lersy et al. 2022 (41)	1.5 T MRI 3 T MRI	T1: acute stage of infection T2: 3 m post-infection T3: 6 m post-infection	March 2020 - May 2020	Hospitalized	Not reported	31 COVID-19	None	Hypoperfusion and hyperperfusion	Subjective + objective	Not reported
Yus et al. 2022 (23)	3 T MRI	T1: 11 m post-infection	February 2021 - September 2021	Hospitalized and non-hospitalized	Excluded	82 long-COVID	None	Hypoperfusion	Objective	Correlation between perfusion abnormalities and olfaction
Callen et al. 2023 (47)	3 T MRI	T1: 8 m post-infection	July 2021 - February 2022	Hospitalized and non-hospitalized	Not reported	15 COVID-19 (7/15 long-COVID)	10 healthy	Lower cerebrovascular reactivity	Subjective	Not reported
Kim et al. 2023 (24)	3 T MRI	T1: 4 m post-infection	May 2020 - September 2021	Non-hospitalized	Excluded	39 COVID-19	11 flu	Hypoperfusion	Subjective + objective	Perfusion abnormalities in COVID-19 patients with fatigue compared to COVID-19 patients without fatigue
Ajčević et al. 2023 (34)	3 T MRI	T1: 6 m post-infection	September 2021 - January 2022	Non-hospitalized	Excluded	24 long-COVID	22 healthy	Hypoperfusion	Subjective + objective	No correlation between perfusion abnormalities and objective cognition (MoCA score). Correlation between perfusion abnormalities and subjective cognition
Ardellier et al. 2023 (35)	3 T MRI	T1: acute stage of infection	February 2020 - May 2020	Hospitalized	Excluded	59 COVID-19	14 healthy	Hypoperfusion and hyperperfusion	Subjective	No correlation between perfusion abnormalities and subjective complaints
Sen et al. 2023 (36)	3 T MRI	T1: post-infection (unspecified)	October 2020 - March 2021	Non-hospitalized	Excluded	28 COVID-19	28 healthy	Hypoperfusion	Objective	Not reported
Díez-Cirarda et al. 2023 (37)	3 T MRI	T1: 11 m post-infection	November 2020 - December 2021	Hospitalized and non-hospitalized	Excluded	84 long-COVID	33 healthy	Hypoperfusion	Subjective + objective	Correlation between hippocampal perfusion and cognition (memory)
Greene et al. 2022 (50)	3 T MRI	T1: 6.5 m post-infection	March 2020 - April 2020	Hospitalized and non-hospitalized	Excluded	22 long-COVID	10 COVID-19 without long-COVID + 60 healthy	BBB leakage	Subjective + objective	No correlation between BBB leakage and objective cognition (MoCA score) or olfaction. Association between BBB leakage and subjective complaints (brain fog)
Shi et al. 2023 (51)	3 T MRI	T1: 3 m post-infection	June 2021 - March 2023	Hospitalized	Not reported	7 COVID-19	17 healthy	BBB leakage	None	–
fMRI										
Scardua Silva et al. 2021 (57)	3 T MRI	T1: 1.5 m post-infection	Not reported	Non-hospitalized	Not reported	87 COVID-19	55 healthy	Reduced functional connectivity	Subjective + objective	Correlation between functional connectivity and fatigue and somnolence
Thunell et al. 2021 (29)	3 T MRI	T1: pre-infection T2: 7 m post-infection	Not reported	Non-hospitalized	Not reported	9 COVID-19	12 healthy	Increased functional connectivity	Objective	Not reported
Fischer et al. 2022 (54)	3 T MRI	T1: acute stage of infection	T1: acute stage of infection	Hospitalized	Excluded	12 COVID-19 with disorder of consciousness	14 healthy + 18 TBI	Reduced functional connectivity	None	–
Esposito et al. 2022 (27)	3 T MRI	T1: 0.5 m post-infection	April 2020 - December 2020	Hospitalized and non-hospitalized	Excluded	27 COVID-19 with hyposmia	18 healthy	Increased segregation within olfactory system	Objective	Correlation between functional connectivity and olfaction in the COVID-19 group
Cattarinussi et al. 2022 (72)	3 T MRI	T1: 4.5 m post-infection	March 2020 - June 2020	Hospitalized and non-hospitalized	Excluded	79 COVID-19	17 healthy	Reduced ReHo and increased ReHo	Subjective	Correlation between ReHo and mental health score
Tsvetanov et al. 2022 (48)	3 T MRI	T1: 6 m post-infection	March 2020 - July 2020	Hospitalized	Included	45 COVID-19	42 healthy	Reduced RSFA	Subjective + objective	Correlation between RSFA and cognition (MoCA), mental health scores, and functional independence (Barthel Index and (inverted) Modified Ranking Scale)
Hafiz et al. 2023 (69)	3 T MRI	T1: 0.5 m post-infection	May 2020 - December 2020	Hospitalized	Excluded	38 COVID-19	31 healthy	Reduced and increased functional connectivity	Subjective	Correlation between functional connectivity and fatigue
Díez-Cirarda et al. 2022 (63)	3 T MRI	T1: 11 m post-infection	November 2020 - December 2021	Hospitalized and non-hospitalized	Excluded	86 long-COVID	36 healthy	Reduced functional connectivity	Subjective + objective	Correlation between functional connectivity and cognition (memory)
Voruz et al. 2023 (67)	3 T MRI	T1: 8.5–9.5 m post-infection	March 2020 - May 2021	Hospitalized and non-hospitalized	Excluded	50 COVID-19	None	Reduced and increased functional connectivity	Subjective + objective	Correlation between functional connectivity and cognition (memory and executive function)
Paolini et al. 2023 (55)	3 T MRI	T1: 6 m post-infection	January 2021 - January 2022	Hospitalized	Excluded	29 long-COVID	29 COVID-19 without long-COVID	Reduced and increased functional connectivity	Subjective	Functional connectivity abnormalities in COVID-19 patients with persisting cognitive complaints compared to patients without persistent complaints
Muccioli et al. 2023 (30)	3 T MRI	T1: 11 m post-infection	April 2020 - December 2020	Non-hospitalized	Excluded	23 COVID-19 with olfactory dysfunction	26 healthy	Increased segregation within olfactory system	Subjective + objective	Correlation with functional connectivity and cognition (memory) and olfaction
Chang et al. 2023 (74)	3 T MRI	T1: 8 m post-infection	February 2021 - February 2022	Hospitalized and non-hospitalized	Excluded	29 long-COVID	21 healthy	Increased neural activation in working memory task	Subjective + objective	Correlation between neural activation and mental health score
Li et al. 2023 (70)	3 T MRI	T1: 6.5 m post-infection	February 2020 - May 2020	Hospitalized	Not reported	35 COVID-19	36 healthy	Decreased and increased neural activity	Subjective	Not reported
Invernizzi et al. 2023 (71)	3 T MRI	T1: pre-infection T2: 1–11 m post-infection	Not reported	Non-hospitalized	Excluded	13 COVID-19	27 healthy	Reduced and enhanced connectivity in regions involved in low-level perceptual learning and memory performance	Objective	Correlation between functional connectivity and cognition (memory)
Díez-Cirarda et al. 2023 (37)	3 T MRI	T1: 11 m post-infection	November 2020 - December 2021	Hospitalized and non-hospitalized	Excluded	84 long-COVID	33 healthy	Reduced connectivity between hippocampal subfield (right head of hippocampus) and regions of the dorsal attention network	Subjective + objective	Not reported
Yulug et al. 2023 (68)	1.5 T MRI	T1: 8 m post-infection	Not reported	Non-hospitalized	Excluded	17 COVID-19	20 healthy	Enhanced connectivity between hippocampal subfield (right hippocampal fissure) and regions of the (para)limbic network	Objective	Correlation between hippocampal functional connectivity and cognition (Alzheimer’s Disease Assessment scale -cognitive)
Churchill et al. 2023 (73)	3 T MRI	T1: 4 m post-infection	May 2020 - December 2021	Non-hospitalized	Excluded	57 COVID-19 (with and without persistent headache)	17 flu	Altered functional brain dynamics in COVID-19 patients with persistent headache	Subjective	Not reported
MRS										
Poletti et al. 2022 (80)	3 T MRI	T1: 4 m post-infection	January 2021 - July 2021	Hospitalized	Excluded	49 COVID-19	none	Reduced glutathione concentration in anterior cingulate cortex with higher white matter hyperintensity load	Subjective	Correlation between glutathione concentration and mental health scores
Ernst et al. 2023 (81)	3 T MRI	T1: 8 m post-infection	February 2021 - February 2022	Hospitalized and non-hospitalized	Excluded	29 long-COVID	25 healthy	Lower total N-acetylaspartate + N-acetylaspartyl-glutamate compounds, frontal white matter glutamate + glutamine, and anterior cingulate cortex myo-inositol levels	Subjective + objective	Correlation between total N-acetylaspartate + N-acetylaspartyl-glutamate compounds and cognition (processing speed, attention, executive function). Correlation between myo-inositol levels and cognition (memory)
[18^F^]-FDG-PET										
Niesen et al. 2021 (75)	PET	T1: 0.5 m post-infection	April 2020 - May 2020	Non-hospitalized	Excluded	11 COVID-19	26 healthy	Hypometabolism and hypermetabolism	Objective	Correlation between metabolic abnormalities and olfaction
Kas et al. 2021 (78)	PET	T1: acute stage of infection T2: 1 m post-infection T3: 6 m post-infection	March 2020 - June 2020	Hospitalized	Included	7 COVID-19 with encephalopathy	23 healthy	Hypometabolism prefrontal and subcortical regions	Subjective + objective	Not reported
Guedj et al. 2021 (21)	PET	T1: 3 m post-infection	May 2020 - September 2020	Hospitalized	Excluded	35 long-COVID	44 healthy	Hypometabolism in prefrontal and temporal regions, thalamus, brainstem, and cerebellum	Subjective	Correlation between metabolic abnormalities and subjective complaints
Sollini et al. 2021 (22)	PET	T1: 4.5 m post-infection	Not reported	Hospitalized	Excluded	13 long-COVID	26 melanoma	Hypometabolism in paralimbic region and thalamus	Subjective	Correlation between metabolic abnormalities and fatigue
Hosp et al. 2021 (46)	PET	T1: 1 m post-infection	June 2020 - January 2021	Hospitalized	Excluded	15 COVID-19	45 healthy	Hypometabolism fronto-parietal regions	Subjective + objective	Correlation between metabolic abnormalities and cognition (MoCA score)
Chammas et al. 2021 (44)	PET	T1: acute stage of infection T2: 1 m post-infection	March 2020 - August 2020	Hospitalized	Not reported	112 COVID-19	25 healthy	Hyperactivation colliculi	None	–
Dressing et al. 2022 (77)	PET	T1: 6.5 m post-infection	June 2020 - January 2021	Hospitalized	Excluded	31 long-COVID	45 healthy	No changes in metabolism	Subjective + objective	No correlation between metabolism and cognition (MoCA score) nor fatigue
Verger et al. 2022 (19)	PET	T1: 10–18 m post-infection	August 2021 - October 2021	Non-hospitalized	Not reported	143 long-COVID	None	Hypometabolism in prefrontal and (para)limbic regions and brainstem	Subjective	Not reported
Lersy et al. 2022 (41)	PET	T1: acute stage of infection T2: 3 m post-infection T3: 6 m post-infection	March 2020 - May 2020	Hospitalized	Not reported	31 COVID-19	None	Hypometabolism in temporal regions, hypermetabolism in colliculi	Subjective + objective	Not reported
Martini et al. 2022 (79)	PET	T1: <1m post-infection T2: 1–3 m post-infection T3: 5–9 m post-infection	October 2020 - November 2021	Hospitalized	Not reported	26 COVID-19	125 healthy	Hypometabolism in prefrontal regions, hypermetabolism in temporal regions and brainstem and cerebellum	Objective	Correlation between metabolic abnormalities and cognition (Mini-Mental State Examination; MMSE)
Goehringer et al. 2023 (20)	PET	T1: 16.5 m post-infection	September 2020 - May 2022	Non-hospitalized	Not reported	28 long-COVID	28 healthy	Hypometabolism in frontal and temporal regions	Subjective + objective	Correlation between metabolic abnormalities and cognition (MoCA score)
Debs et al. 2023 (76)	PET	T1: pre-infection T2: 0–2 m post-infection T3: 2–6 m post-infection T4: 6–12 m post-infection	April 2020 - October 2021	Hospitalized and non-hospitalized	Excluded	45 long-COVID	52 healthy	Hypometabolism and hypermetabolism	Subjective	Correlation between metabolic abnormalities and subjective complaints
[18^F^]-FEPPA-PET										
Braga et al. 2023 (82)	PET	T1: 0–6 m post-infection T2: 7–24 m post-infection	April 2021 - June 2022	Non-hospitalized	Excluded	20 long-COVID	20 healthy	Elevated gliosis in subcortical regions	Subjective	Correlation between gliosis and cognition (motor speed)


Abbreviations: DTI = diffusion tensor imaging, ASL = arterial spin labelling, DSC-MRI = dynamic susceptibility contrast magnetic resonance imaging, DCE-MRI = dynamic contrast-enhanced magnetic resonance imaging, fMRI = functional magnetic resonance imaging, MRS = magnetic resonance spectroscopy, [18^F^]-FDG-PET = fluorodeoxyglucose positron emission tomography; [18^F^]-FEPPA-PET = [18^F^]-N-(2-(2-Fluoroethoxy)benzyl)-N-(4-phenoxypyridin-3-yl) positron emission tomography, FA = fractional anisotropy, MD = mean diffusivity, RD = radial diffusivity, AD = axial diffusivity, FW = free water, ODI = orientation dispersion index, V_ic_ = volume of intracellular water compartment, V_iso_ = volume of isotropic diffusion compartment, FC = fiber cross-section, FD = fiber density, FDC = fiber density and cross-section, BBB = blood–brain barrier, ReHo = regional homogeneity, RSFA = resting-state fluctuation amplitude, MoCA = Montreal Cognitive Assessment.

a Note that multiple imaging timepoints can either indicate a cross-sectional or longitudinal design.

b Subjective neuropsychological complaint measurements include self-reported symptoms and complaints (e.g., fatigue or mental health scores) acquired through clinical interviews or questionnaires. Objective neuropsychological complaint measurements include neuropsychological test battery (e.g., assessment of cognitive domains, such as memory and executive functioning) or olfaction.

### 3.1 Olfactory system abnormalities

Due to its potential pathway for the SARS-CoV-2 virus to enter the brain and many COVID-19 patients presenting with olfactory dysfunction, the olfactory system has received great interest in COVID-19 research. Structural MRI revealed abnormalities, including signal abnormalities and volume changes, particularly in the olfactory bulb. Advanced neuroimaging techniques have focused on these findings in the olfactory bulb and have expanded the region of interest to brain regions involved in the olfactory system. In patients with the long-COVID syndrome, hypometabolism was observed using [18^F^]-FDG-PET imaging in the bilateral olfactory bulbs (Verger et al., 2022), but also in other regions of the olfactory system, including the orbitofrontal cortex (Verger et al., 2022, Goehringer et al., 2023, Guedj et al., 2021, Sollini et al., 2021) and parahippocampal gyrus (Goehringer et al., 2023, Sollini et al., 2021). In line with these findings, hypoperfusion has been observed using arterial spin labelling (ASL) in regions of the olfactory system in long-COVID patients (Yus et al., 2022, Kim et al., 2023). In recovered COVID-19 patients with persistent olfactory dysfunction, higher mean diffusivity (MD) was observed in orbitofrontal white matter tracts compared to recovered COVID-19 patients without olfactory dysfunction (Campabadal et al., 2023). Similarly, Douaud et al. (Douaud et al., 2022) observed a longitudinal increase (pre-COVID versus post-COVID) in MD in the orbitofrontal cortex in COVID-19 patients, which was accompanied by reduced grey matter thickness in the orbitofrontal cortex and parahippocampal gyrus. In the absence of significant abnormalities on structural clinical imaging, a structural connectivity study in recently recovered COVID-19 patients with olfactory dysfunction reported higher connectivity (indicated by a connectivity index reflecting the ratio between streamlines reaching target areas and generated streamlines from the seed region) within the olfactory system compared to healthy controls (Esposito et al., 2022). Another structural connectivity study reported reduced integration and increased segregation within the olfactory system (Bispo et al., 2023). Assessment using resting-state fMRI revealed increased functional connectivity within the olfactory system in long-COVID patients (Thunell et al., 2022), with more elaborate studies reporting increased segregation of regions within the olfactory system (Esposito et al., 2022, Muccioli et al., 2023), in line with the structural connectivity study by Bispo et al. (Bispo et al., 2023). Interestingly, structural clinical MRI measures that were also obtained in these connectivity studies failed to distinguish COVID-19 patients from healthy controls (Esposito et al., 2022, Thunell et al., 2022, Muccioli et al., 2023).

### 3.2 Cerebrovascular malfunction

Using standard clinical imaging sequences sensitive to vascular changes, such as SWI, it became apparent that microbleeds are common in the COVID-19 brain (Agarwal et al., 2020, Hernández-Fernández et al., 2020, Conklin et al., 2021). However, SWI is a static imaging technique and is therefore not sensitive to dynamic processes, such as cerebral perfusion. Imaging techniques for perfusion measurement include ASL and dynamic susceptibility contrast MRI (DSC-MRI). Tracking blood flow over time, either by magnetically labelling blood (ASL) or by using a contrast agent (DSC-MRI), can provide information on cerebral blood flow (CBF) and cerebral blood volume (CBV).

**Fig. 4:**
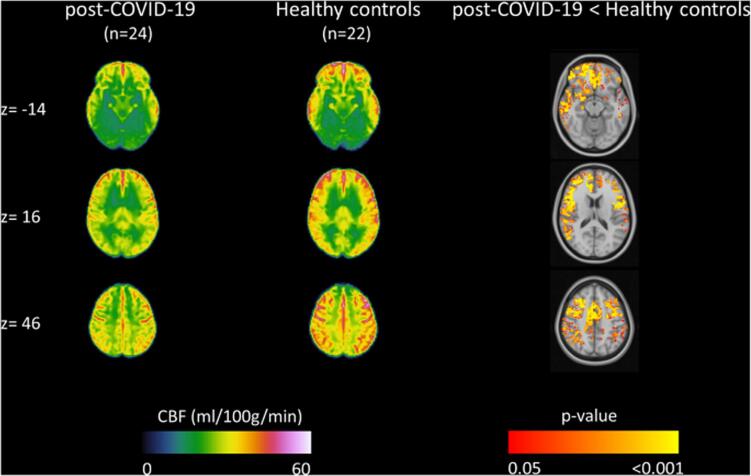
**Cortical hypoperfusion pattern in patients with long-COVID syndrome.** Group averages of arterial spin labelling cerebral blood flow (ml/100 g/min) maps at different slice positions (z) in the long-COVID (left) and healthy control (middle) groups. A significant hypoperfusion pattern was observed in long-COVID patients six months after the infection compared to healthy controls in predominantly frontal, temporal, and parietal regions (right). Adapted with permission from Ajčević et al. (34).

Most studies assessing perfusion in COVID-19 patients report lower global tissue perfusion, i.e., hypoperfusion (Kim et al., 2023, Ajčević et al., 2023, Ardellier et al., 2023, Sen et al., 2023, Díez-Cirarda et al., 2023, Chougar et al., 2020, Henry-Feugeas et al., 2020, Lambrecq et al., 2021, Lersy et al., 2022, Qin et al., 2021, Tian et al., 2022). Hypoperfusion is typically more severe in patients with severe COVID-19 compared to mild COVID-19 (Qin et al., 2021). Some studies have additionally observed hyperperfusion in a minority of their COVID-19 patient sample (Ardellier et al., 2023, Henry-Feugeas et al., 2020, Lambrecq et al., 2021, Lersy et al., 2022, Chammas et al., 2021). Two studies observed neither hypoperfusion nor hyperperfusion within COVID-19 patients (Klironomos et al., 2020) or between COVID-19 patients and healthy controls (Hosp et al., 2021). Perfusion abnormalities are observed globally, predominantly in frontal and temporal cortical regions (Ajčević et al., 2023, Ardellier et al., 2023, Lambrecq et al., 2021, Lersy et al., 2022, Qin et al., 2021, Tian et al., 2022), but also in parietal cortical regions (Ajčević et al., 2023, Ardellier et al., 2023, Henry-Feugeas et al., 2020, Qin et al., 2021), occipital cortical regions (Henry-Feugeas et al., 2020, Qin et al., 2021), subcortical regions (Kim et al., 2023, Díez-Cirarda et al., 2023, Qin et al., 2021), midbrain (i.e., colliculi) (Chammas et al., 2021), cerebellum (Ardellier et al., 2023), and in the white matter (Sen et al., 2023) (Fig. 4). Serial perfusion imaging revealed (partial) CBF recovery 5–10 months after initial infection in patients with (Lersy et al., 2022) or without persisting complaints (Tian et al., 2022). However, other serial studies still observed lower CBF values in COVID-19 patients compared to healthy controls 3–7 months after the infection (Kim et al., 2023, Ajčević et al., 2023, Qin et al., 2021). Importantly, perfusion abnormalities have been reported even in the absence of structural abnormalities (Ajčević et al., 2023, Ardellier et al., 2023, Sen et al., 2023).

Cerebrovascular reactivity (CVR), or cerebral autoregulation (CA), is another functional measure that can be extracted from perfusion imaging data. Where Callen et al. (Callen et al., 2023) did not observe significant differences in global CBF measures between recovered COVID-19 patients and healthy controls, CVR was globally impaired in COVID-19 patients around eight months after the initial infection. Here, CVR was assessed using the absolute increase in CBF between two ASL acquisitions, occurring after administering a cerebral vasodilator. Furthermore, within the COVID-19 patient group, CVR was diminished in patients with long-COVID syndrome, although this finding was not significant. Moreover, another measure thought to reflect vasoregulation is the resting-state fluctuation amplitude (RSFA), which can be extracted from resting-state functional MRI data. Significantly lower RSFA has been observed in temporal-parietal regions in COVID-19 patients around six months after hospital discharge, compared to healthy controls (Tsvetanov et al., 2022). Widespread changes in fronto-parietal RSFA were related to the severity of the COVID-19 infection.

### 3.3 Blood-brain barrier leakage

The blood–brain barrier (BBB) has been considered a target of interest regarding a potential point of entry for the SARS-CoV-2 virus into brain tissue. The most widely used neuroimaging technique in humans to assess BBB integrity is dynamic contrast-enhanced MRI (DCE-MRI). Here, a contrast agent (Gadolinium: Gd-DTPA) is injected intravenously, which would normally not cross an intact BBB, and 'leakage' of this contrast agent out of the blood vessels into the brain tissue can be quantified. This results in a measure of the permeability of the BBB, with higher permeability indicating worse integrity of the BBB. Non-contrast alternatives to quantify BBB leakage based on the ASL technique are, however, emerging (Lin et al., 2018).

To this date, BBB imaging studies in COVID-19 patients are sparse. However, Greene et al. (Greene et al., 2024) used DCE-MRI to assess BBB integrity in patients with long-COVID syndrome. DCE-MRI revealed increased whole-brain leakage of the BBB in long-COVID patients with complaints of brain fog compared to completely recovered COVID-19 patients and long-COVID patients without complaints of brain fog. Structural clinical imaging did not reveal pathology in any of these patients. Another study assessed BBB integrity in COVID-19 intensive care unit (ICU) survivors approximately three months after the infection using water-extraction-with-phase-contrast-arterial-spin-tagging (WEPCAST) (Shi et al., 2023). In comparison to healthy controls, increased whole-brain BBB leakage was observed in COVID-19 ICU survivors, whereas no structural or perfusion abnormalities were apparent.

### 3.4 White matter microstructural integrity alterations

Structural clinical imaging revealed that the cerebral white matter is extensively damaged in COVID-19, i.e., abundant white matter hyperintensities and microbleeds. This was most prominent in hospitalized patients during the acute stage of the infection. A versatile advanced neuroimaging technique to assess the microarchitecture of cerebral white matter and extract white matter tract-specific properties is diffusion tensor imaging (DTI). Measures to quantify the microarchitecture include fractional anisotropy (FA), MD, radial diffusivity (RD), and axial diffusivity (AD). With the advent of extended DTI models, other measures, such as the free-water fraction, are now available, providing additional information on intra- and extracellular tissue and fluid compartments (Pasternak et al., 2009). Moreover, with the option to estimate the configuration of white matter bundles (i.e., tractography), tract-specific measures can be extracted, including fiber density and tract volume and length.

**Fig. 5:**
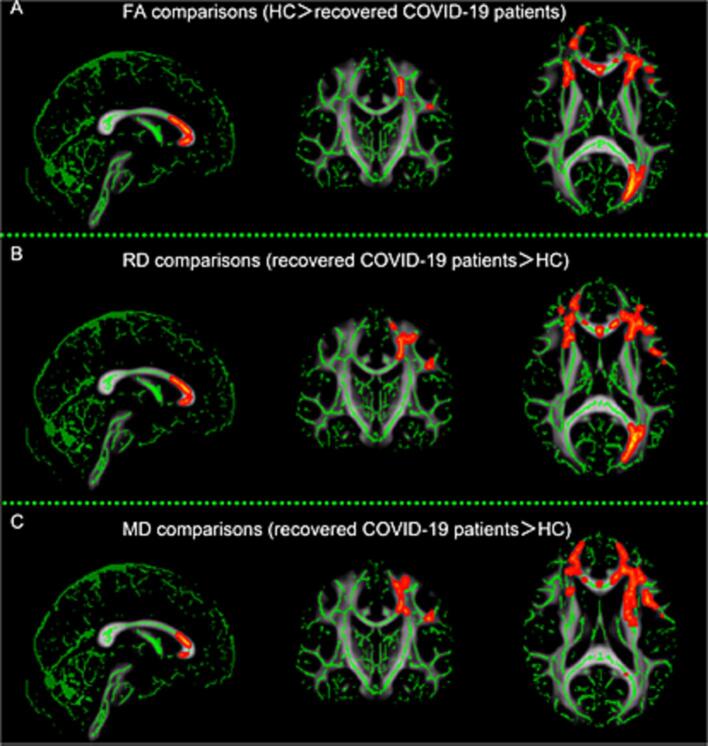
**Widespread white matter microstructural alterations in recovered COVID-19 patients.** Diffusion tensor imaging revealed lower fractional anisotropy (FA) in recovered COVID-19 patients three months after the infection compared to healthy controls in multiple brain regions (A). These changes were accompanied by increased radial diffusivity (B) and increased mean diffusivity (MD) (C). Reprinted with permission from Yang et al. (58).

The majority of DTI studies in COVID-19 reported lower FA in COVID-19 patients compared to healthy controls (Qin et al., 2021, Bispo et al., 2022, Fischer et al., 2022, Paolini et al., 2023, Planchuelo-Gómez et al., 2023, Silva et al., 2021, Yang et al., 2021), with lower FA in ICU compared to non-ICU COVID-19 patients (Huang et al., 2022). Considering whether these abnormalities are specific to COVID-19, it is interesting to note that FA values in COVID-19 patients were comparable to patients with traumatic brain injury (TBI) (Fischer et al., 2022). In line with lower FA values, most DTI studies reported higher MD (Bispo et al., 2022, Paolini et al., 2023, Planchuelo-Gómez et al., 2023, Silva et al., 2021, Yang et al., 2021, Petersen et al., 2023) and higher RD (Bispo et al., 2022, Paolini et al., 2023, Planchuelo-Gómez et al., 2023, Silva et al., 2021, Yang et al., 2021) in COVID-19 patients compared to controls. These microstructural changes are reported widely throughout the brain – affecting association, commissural, projection, and subcortical white matter fiber bundles – and suggest less restricted diffusion, indicative of demyelination of these white matter tracts (Fig. 5). Other DTI measures have further supported less restricted diffusion, including increased extracellular free water (Petersen et al., 2023, Huang et al., 2023) and correlated diffusion imaging (Teller et al., 2023). However, Teller et al. (Teller et al., 2023) reported more restricted diffusion in the cerebellum. Furthermore, axonal injury and less coherent orientation of axons have been reported, as indicated by lower AD (Planchuelo-Gómez et al., 2023, Díez-Cirarda et al., 2022), lower fiber density (Bispo et al., 2022, Lathouwers et al., 2023), and lower volume fraction of intracellular water (Huang et al., 2022). Other tract properties reveal lower tract volume (Qin et al., 2021, Tian et al., 2022) and tract length (Qin et al., 2021) in association, commissural, projection, and subcortical white matter fiber bundles. Strikingly, most of these microstructural changes have been observed after recovery from the initial infection, ranging from 2 to 24 months post-infection. However, some serial studies showed that these microstructural changes can be dynamic and have the potential to recover over time (Tian et al., 2022, Huang et al., 2023, Teller et al., 2023, Zhang et al., 2023).

### 3.5 Structural and functional brain network alterations

The assessment of the integrity of individual or whole-brain white matter bundles is complemented by quantifying the efficiency of information transmission within the brain. This can be approached using multiple strategies on a regional or network level. For instance, DTI can be used to assess structural connectivity, i.e., a connectivity measure based on the anatomical white matter connections between brain regions. Consequently, structural connectivity measures allow quantification of various brain network measures reflecting how efficiently a certain network behaves. Another approach is to study brain function over time using fMRI, either task-based fMRI or resting-state fMRI. Task-based fMRI is commonly used to study changes in the blood-oxygen-level-dependent (BOLD) signal (a proxy for neural activity) in response to a certain stimulus. In contrast, resting-state fMRI is used to study the synchronicity of spontaneous BOLD signal fluctuations (i.e., in the absence of a stimulus), allowing the quantification of functional connectivity within the brain.

The efficiency of brain networks in terms of structural connectivity has not been extensively studied in COVID-19. Yang et al. (Yang et al., 2021) used DTI to determine global network properties parameters in recovered COVID-19 patients three months after hospital discharge. The authors reported significantly lower global efficiency and longer shortest path length in recovered COVID-19 patients compared to healthy controls, indicating impaired capacity of information transfer in recovered COVID-19 patients. In a study by Tassignon et al. (Tassignon et al., 2023), previously hospitalized COVID-19 patients were assessed 1–2 months and 3–4 months after hospital discharge. A decrease in characteristic path length was observed between the two time points, suggesting improved efficiency of information transmission over time.

Resting-state fMRI has been more widely used in COVID-19 research, particularly in terms of functional connectivity. Depending on the brain regions involved, functional connectivity findings in COVID-19 patients range from enhanced to reduced connectivity. In the absence of substantial structural damage, distinct patterns of enhanced and reduced functional connectivity were observed when comparing mild, moderate, and severe COVID-19 patients around nine months after the infection (Voruz et al., 2023). More specifically, the authors reported reduced connectivity in the attention, salience, and sensorimotor networks in severely affected patients compared to mildly and moderately affected patients. In the same study, severely affected patients exhibited enhanced functional connectivity in the default mode network and subcortical network compared to mildly affected patients. Comparing moderately and mildly affected patients revealed enhanced connectivity in moderately affected patients, implicating the subcortical, sensorimotor, cerebellar, and temporal-parietal networks. The authors suggested that enhanced connectivity may result from a compensatory mechanism. In another study, enhanced connectivity was observed in long-COVID patients compared with recovered COVID-19 patients in regions of the salience, attention, and sensorimotor networks (Paolini et al., 2023). This study additionally reported reduced functional connectivity in the long-COVID group in the default mode and frontoparietal networks. Connectivity to/from and within subfields of the hippocampus has received specific attention in COVID-19 patients. Reduced connectivity between the right head of hippocampus and regions of the dorsal attention network was observed in non-hospitalized patients with long-COVID compared to healthy controls approximately eleven months after the infection (Díez-Cirarda et al., 2023). In contrast, another study reported enhanced connectivity between subfields of the hippocampus and other regions of the (para)limbic network in recovered COVID-19 patients (Yulug et al., 2023).

Functional connectivity in hospitalized COVID-19 patients at different stages of recovery has been compared to healthy controls. In the acute stage of the infection, reduced functional connectivity within the default mode network and between the default mode network and salience network was reported in COVID-19 patients with reduced consciousness compared to healthy controls (Fischer et al., 2022). Another study assessed functional connectivity two weeks after hospital discharge and reported reduced functional connectivity in cerebellar regions but enhanced functional connectivity in regions of the subcortical and default mode network (Hafiz et al., 2023). Approximately six months after hospital discharge, reduced functional connectivity was observed in recovered COVID-19 patients compared to healthy controls, affecting the visual, paralimbic, attention, sensorimotor, and default mode networks (Li et al., 2023). Furthermore, Diez-Cirarda et al. (Díez-Cirarda et al., 2022) observed reduced functional connectivity between limbic, paralimbic, and cerebellar regions in hospitalized and non-hospitalized patients close to one year after the infection. A study in non-hospitalized patients observed a trend towards reduced functional connectivity affecting the salience and visual network around two months after the infection (Silva et al., 2021). Another study in non-hospitalized patients assessed changes in functional connectivity after a SARS-CoV-2 infection with pre-COVID data and demonstrated that functional connectivity involving prefrontal and limbic structures was more drastically reduced in COVID-19 patients than in healthy controls (Invernizzi et al., 2023). Conversely, an increase in functional connectivity was observed in regions involved in low-level perceptual learning processes.

In addition to functional connectivity throughout the brain, resting-state fMRI can be used to extract local fMRI metrics, such as regional homogeneity (ReHo) and amplitude of low-frequency fluctuation (ALFF), which reflect synchronicity within a region and spontaneous neural activity, respectively. In recovered COVID-19 patients, ReHo was reduced (i.e., less synchronous) within regions of the language and executive network but enhanced in the hippocampus (Cattarinussi et al., 2022). Another study, focused on recovered COVID-19 patients around six months after hospital discharge, observed in comparison to healthy controls an increased ALFF (i.e., hyperactivity) in regions of the visual, language, and default mode network but decreased ALFF (i.e., hypoactivity) in regions of the paralimbic and executive network (Li et al., 2023). A study in non-hospitalized COVID-19 patients four months after the infection observed altered functional brain dynamics – here characterized by the Hurst component, which is typically suppressed during physiological and psychological distress – in patients with persistent headache compared to patients without headache and healthy controls (Churchill et al., 2023). Altered functional brain dynamics were particularly apparent in temporal, sensorimotor, and insular brain regions, and were accompanied by reduced functional connectivity and BOLD activity.

A single study used task-based fMRI, revealing greater activation during a working memory task in regions associated with working memory in long-COVID patients around eight months after the infection, compared to healthy controls (Chang et al., 2023). The increase in activation coincided with increased activation of the superior frontal gyrus (a region of the attention network) and less deactivation of the posterior cingulate gyrus (a region of the default mode network).

### 3.6 Changes in metabolic activity

Neuroimaging modalities other than MRI have a unique potential to provide new insights into COVID-19 metabolic and inflammatory pathology. Depending on the radiotracer, PET imaging can be used to assess physiological function, including different metabolic processes. For instance, [18^F^]-FDG-PET imaging is sensitive to the regional uptake of glucose and serves as a global marker for altered metabolic activity. On the other hand, [18^F^]-FEPPA-PET is sensitive to microglial activation and, therefore, serves as a marker for inflammation.

**Fig. 6:**
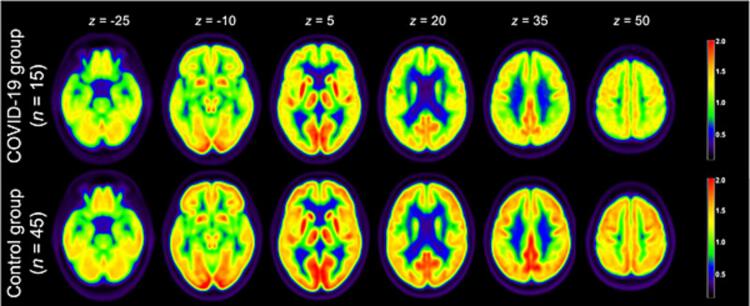
**COVID-19 hypometabolism pattern.** Group averages of [^18^F]-FDG-PET scans at different slice positions (z) of the recovered COVID-19 group (top row) and healthy control group (bottom row). [^18^F]-FDG-PET revealed a hypometabolism pattern in the COVID-19 group one month after the infection, particularly affecting frontal, temporal, and parietal brain regions. Adapted with permission from Hosp et al. (46).

The majority of studies using [^18^F]-FDG-PET imaging observed altered patterns of glucose metabolism in the COVID-19 brain (Verger et al., 2022, Goehringer et al., 2023, Guedj et al., 2021, Sollini et al., 2021, Lersy et al., 2022, Chammas et al., 2021, Hosp et al., 2021, Niesen et al., 2021, Debs et al., 2023, Dressing et al., 2022, Kas et al., 2021, Martini et al., 2022) and also in patients without visible abnormalities on structural MRI (Kas et al., 2021). These studies have revealed a seemingly characteristic pattern of hypometabolism in COVID-19, predominantly involving frontal, parietal, and temporal brain regions (Fig. 6). However, the whole brain is affected, as hypometabolism has also been reported in other regions, including limbic regions (Verger et al., 2022, Guedj et al., 2021, Sollini et al., 2021, Kas et al., 2021), the brainstem (Verger et al., 2022, Guedj et al., 2021), and the cerebellum (Verger et al., 2022, Guedj et al., 2021, Niesen et al., 2021). Conversely, hypermetabolism has also been observed in (para)limbic regions, the brainstem, the cerebellum (Niesen et al., 2021, Debs et al., 2023, Martini et al., 2022), and also in the colliculi (Lersy et al., 2022, Chammas et al., 2021). Where most of these abnormalities were relative to [18^F^)-FDG-PET scans of healthy control subjects, Debs et al. (Debs et al., 2023) compared post-infection and pre-infection [18^F^]-FDG-PET scans within subjects and showed both hypometabolic and hypermetabolic alterations. Altered glucose metabolism has been observed during the acute infection (Chammas et al., 2021, Hosp et al., 2021, Niesen et al., 2021, Kas et al., 2021, Martini et al., 2022) but also months after recovery (Lersy et al., 2022, Chammas et al., 2021, Debs et al., 2023, Kas et al., 2021, Martini et al., 2022) and in long-COVID patients (Verger et al., 2022, Goehringer et al., 2023, Guedj et al., 2021, Sollini et al., 2021). However, some results suggest normalization of these metabolic abnormalities, with studies observing subsiding or absence of any alterations (Dressing et al., 2022, Martini et al., 2022).

In addition to PET, MR spectroscopy provides a window into metabolic activity. Using MR spectroscopy, Poletti et al. (Poletti et al., 2022) showed that lower levels of glutathione (the main antioxidant in the brain) in the anterior cingulate cortex were related to a higher global white matter hyperintensity (WMH) load within long-COVID patients. Lower levels of glutathione suggest that the brain is more vulnerable to inflammation and oxidative stress. Another MR spectroscopy study revealed lower total N-acetyl-compounds (N-acetylaspartate + N-acetylaspartyl-glutamate), lower glutamate + glutamine levels in the frontal white matter, and lower myo-inositol levels in the anterior cingulate cortex in long-COVID patients eight months after the infection compared to healthy controls (Ernst et al., 2023). These abnormal compound levels are indicative of neuronal injury and glial dysfunction.

In a [18^F^]-FEPPA PET study focusing on inflammatory changes, Braga et al. (Braga et al., 2023) observed that gliosis (a process involving the proliferation of glial cells) was elevated in the basal ganglia (and a trend towards significant elevation in the anterior cingulate cortex, thalamus, and insula) in long-COVID patients, suggesting ongoing inflammation months after the initial infection.

### 3.7 Postmortem findings in COVID-19 decedents

#### 3.7.1 Imaging of postmortem COVID-19 brain tissue

Brain imaging of postmortem brain tissue has been performed in two studies. Coolen et al. (Coolen et al., 2020) used 3 T MRI to study structural brain abnormalities in 19 deceased hospitalized COVID-19 patients. The brainstem was of particular interest due to its regulatory role in respiration and low perceived dyspnea in COVID-19 patients in the acute phase, but no abnormalities were found in this region. The bilateral olfactory bulbs were found to be asymmetric in 4 patients, but downstream olfactory tract abnormalities were not found. Other abnormalities were subcortical microbleeds and macrobleeds in two patients, edematous changes indicative of posterior reversible encephalopathy syndrome in one patient, and nonspecific deep white matter changes (e.g., white matter hyperintensities (WMH)) in another patient. Another study using structural MRI on postmortem brain material of 20 COVID-19 ICU patients observed microbleeds, WMHs, and enlarged perivascular spaces (Agrawal et al., 2022). In this study, MRI was complemented with histology, which revealed vascular damage (i.e., ischemic lesions and hemorrhage), hypoxic changes (i.e., neuronal shrinkage, red neurons, pyknotic neurons), inflammation (i.e., microglia activation, neuronophagia), lymphocytic inflammation, and pathology related to neurodegenerative disease. These changes were found throughout the brain, affecting cortical and subcortical regions, the brainstem, cerebellum, olfactory bulb, blood vessels, and meninges.

#### 3.7.2 Histological findings in postmortem COVID-19 brain tissue

The remaining 16 postmortem studies were not combined with clinical imaging techniques. Macroscopy revealed an edematous brain surface with widened gyri, flattened surface, narrowed sulci, and leptomeningeal vessel congestion (Fabbri et al., 2022), and macro- and microbleeds (Santana et al., 2021). Microscopic histology and immunohistochemistry revealed extensive vascular damage, including microthrombi (Fabbri et al., 2022, Santana et al., 2021, Lee et al., 2022), ischemic damage (Matschke et al., 2020, Fabbri et al., 2022, Gelpi et al., 2023, Normandin et al., 2023, Thakur et al., 2021, Zhang et al., 2023), and hemorrhage (Normandin et al., 2023, Zhang et al., 2023). Furthermore, inflammatory processes were characterized by microglia and astrocyte activation (Matschke et al., 2020, Agrawal et al., 2022, Fabbri et al., 2022, Gelpi et al., 2023, Thakur et al., 2021, Zhang et al., 2023, Ruz-Caracuel et al., 2022, Schwabenland et al., 2021), predominantly in the brainstem and cerebellum, and in some studies also in the olfactory bulb (Matschke et al., 2020, Agrawal et al., 2022), basal ganglia (Matschke et al., 2020), and white matter (Thakur et al., 2021). In addition, cytotoxic T lymphocyte cells have been found in blood vessels (Agrawal et al., 2022, Fabbri et al., 2022, Thakur et al., 2021), perivascular spaces (Lee et al., 2022, Zhang et al., 2023, Schwabenland et al., 2021, Colombo et al., 2021), meninges (Matschke et al., 2020, Agrawal et al., 2022, Thakur et al., 2021), brain parenchyma (Agrawal et al., 2022, Santana et al., 2021, Normandin et al., 2023, Colombo et al., 2021), brainstem (Matschke et al., 2020, Colombo et al., 2021), and cerebellum (Matschke et al., 2020, Colombo et al., 2021). BBB damage (Zhang et al., 2023, Schwabenland et al., 2021) and microvascular endothelitis (Rhodes et al., 2021) have also been reported. Some studies have focused specifically on the olfactory system (Ho et al., 2022, Khan et al., 2022, Khan et al., 2021). For instance, Ho and colleagues (Ho et al., 2022) reported lower axon density and more microvascular damage in the olfactory bulbs and tracts in COVID-19 compared to non-COVID-19 decedents. However, two studies by Khan and colleagues (Khan et al., 2022, Khan et al., 2021) only observed damage to the olfactory mucosa, whereas the olfactory bulb and brain parenchyma were spared.

Beach and colleagues (Beach et al., 2021) reported that acute or subacute axonal damage, using positive β-amyloid precursor protein (APP) white matter staining, was more severe in patients dying from COVID-19 compared to the control group consisting of non-COVID-19 pneumonia and non-pneumonia patients. However, the COVID-19 decedents did not have more severe axonal damage than the non-COVID-19 pneumonia control patients alone. Multiple other studies also did not observe damage specific to COVID-19, as the histological findings were typically age-related (Eschbacher et al., 2022, Serrano et al., 2021), were similar to abnormalities observed in non-COVID-19 severely ill patients (Gelpi et al., 2023), and corresponded with sequelae of critical illness or treatment (Gelpi et al., 2023, Normandin et al., 2023, Eschbacher et al., 2022). These studies did not find clear evidence of viral infection of the brain, which is in line with many studies failing to detect viral protein in most COVID-19 decedents (Agrawal et al., 2022, Fabbri et al., 2022, Gelpi et al., 2023, Normandin et al., 2023, Ruz-Caracuel et al., 2022, Khan et al., 2022, Khan et al., 2021, Eschbacher et al., 2022, Serrano et al., 2021). However, viral protein has been found in the brainstem (Matschke et al., 2020) and viral antigen has been detected in cells expressing the ACE2-receptor – a receptor known for the SARS-CoV-2 virus to bind (Beyerstedt et al., 2021) – in the vascular compartment (Schwabenland et al., 2021).

## 4 Discussion

This scoping review assessed the merits of advanced neuroimaging techniques as applied in COVID-19 patients, relative to structural clinical imaging and postmortem assessment (i.e., histology). Neuroimaging in COVID-19 is primarily dominated by MRI (n = 60 studies) and PET (n = 13 studies). Advanced neuroimaging was mainly applied in hospitalized patients above fifty years of age, ranging from imaging in the acute stage of the infection until two years after the infection. Even in the absence of any anatomical abnormalities on structural clinical imaging, advanced neuroimaging revealed widespread perfusion abnormalities (ASL and DSC-MRI), BBB leakage (DCE-MRI and WEPCAST), white matter alterations (DTI), altered structural and functional connectivity (DTI and fMRI), and metabolic abnormalities (MR spectroscopy and PET). On a microscopic level, postmortem assessment has similarly revealed damage throughout the brain, including damage to the neurovascular unit, BBB, and white matter fibers. However, no unified COVID-19-specific imaging pattern regarding localization or type of cerebral damage could be subtracted, as the abnormalities are diverse and widely spread throughout the brain.

### 4.1 Neuroimaging and neuropathological processes of COVID-19

Several mechanisms of action of how the brain is affected by COVID-19 have been previously hypothesized (Crook et al., 2023). These mechanisms include pathways of direct viral infection of brain tissue – raising the question of how the virus enters the brain tissue – and indirect infection, for example, through systemic inflammation or vascular pathology. The idea of the olfactory bulb as a hub for viral entry into the brain has received great attention. Advanced neuroimaging studies have demonstrated that multiple facets of the olfactory system are affected, either locally or on a network level. Although it has not been exclusively established if the SARS-CoV-2 virus directly causes damage in the olfactory system using neuroimaging, these findings show the unequivocal implication of the olfactory system in COVID-19, possibly explaining olfactory dysfunction during and after recovery of the initial infection. Alternatively, it has been suggested that the virus could be transported via the bloodstream and cross the BBB, a previously reported capability in mice (Rhea et al., 2021). Indeed, evidence of BBB impairment has been reported in humans (Shi et al., 2023, Campbell et al., 2022), although this damage could also be related to inflammatory processes. With most postmortem studies reporting an absence of viral protein in the CSF or brain tissue, the likelihood of direct infection is decreasing.

Conversely, support for indirect neural damage is increasing. Multiple pathophysiological processes are likely at play with the various cerebral abnormalities reported in COVID-19. Largescale white matter alterations and perfusion abnormalities may result from hypoxic injury and inflammation, affecting brain function on a network level. Although structural clinical imaging have already hinted towards vascular and inflammatory processes at play (e.g., stroke, hemorrhage, microbleeds, white matter hyperintensities, and enlarged perivascular spaces), advanced neuroimaging techniques have expanded this knowledge by providing insight into how cerebral damage affects brain function, in terms of cerebrovascular functioning (e.g., hypoperfusion and BBB leakage), local and global brain connectivity (e.g., enhanced or reduced structural and functional connectivity) and metabolic activity (e.g., hypometabolism). Histology of postmortem material has provided additional microscopic evidence for damage to the neurovascular unit, inflammatory damage and neuronal and axonal injury. These findings yield mechanistic insight into the cellular processes that underlie COVID-19 pathology as visualized using in vivo advanced neuroimaging techniques.

Moreover, several studies demonstrated that advanced neuroimaging techniques are sensitive to cerebral abnormalities, even without significant findings using structural clinical imaging (Esposito et al., 2022, Ajčević et al., 2023, Ardellier et al., 2023, Sen et al., 2023, Voruz et al., 2023, Kas et al., 2021). This increase in sensitivity also offers the potential to reveal more subtle damage, which can be relevant to reach a broader target group (i.e., mildly infected COVID-19 patients and patients with long-COVID syndrome) or to gain insight into pathological processes preceding irreversible damage. Indeed, many advanced neuroimaging studies have reported cerebral damage in patients with long-COVID syndrome who had not been hospitalized during the infection (Verger et al., 2022, Goehringer et al., 2023, Yus et al., 2022, Ajčević et al., 2023, Díez-Cirarda et al., 2023, Callen et al., 2023, Díez-Cirarda et al., 2022, Chang et al., 2023, Debs et al., 2023, Ernst et al., 2023, Braga et al., 2023).

### 4.2 COVID-19-specificity of neuroimaging findings

Due to the heterogeneity of the types and spatial distribution of cerebral abnormalities in the COVID-19 brain, it is challenging to recognize a pattern specific for COVID-19 brain damage. Many PET studies refer to the 'COVID-19 hypometabolism pattern', typically recognized in COVID-19 patients and predominantly involving prefrontal, parietal, and limbic regions (Hosp et al., 2021, Niesen et al., 2021, Kas et al., 2021). In line with these findings, perfusion abnormalities were commonly found in overlapping regions. However, in addition to these widely affected gray matter regions, numerous studies report on extended COVID-19-affected brain areas, implicating many white matter tracts. Thus, capturing the full spectrum of cerebral abnormalities specific to COVID-19 remains challenging.

Unfortunately, COVID-19 advanced imaging studies with a control group of non-COVID-19 patients – such as those with hypoxic respiratory failure, sepsis, influenza, or common flu – are lacking. Conklin et al. (Conklin et al., 2021) previously described a distribution of lesions on SWI MRI images in critically ill COVID-19, which was found to be similar in patients with hypoxic respiratory failure, sepsis, and disseminated intravascular coagulation. From our search results, only four studies compared their findings in COVID-19 to other patient groups (Sollini et al., 2021, Kim et al., 2023, Fischer et al., 2022, Churchill et al., 2023), with one showing similarities to patients with traumatic brain injury (TBI) in terms of white matter alterations (Fischer et al., 2022). However, compared to COVID-negative patients with flu-like symptoms, COVID-19 patients do show perfusion abnormalities (Kim et al., 2023) and altered functional brain activity (Churchill et al., 2023). Moreover, it has been suggested that many COVID-19 patients present with unrelated comorbid findings of neurodegeneration and cerebrovascular disease (Eschbacher et al., 2022), which may obscure reported findings. This also complicates the identification of COVID-19-specific biomarkers.

### 4.3 Role of neuroimaging in neurological disease progression of COVID-19

A major strength of neuroimaging is that it can be used repeatedly over time with a limited number of adverse events. Hence, longitudinal brain imaging can be used to monitor the dynamics of neurological complications over time. Since the long-term effects of COVID-19 are becoming more apparent, the importance of long-term and longitudinal brain imaging is emphasized. Although the number of serial studies in humans is sparse, some evidence shows potential for (partial) normalization of cerebral abnormalities over time (Tian et al., 2022, Huang et al., 2023, Zhang et al., 2023, Dressing et al., 2022, Martini et al., 2022). However, since many studies reported cerebral abnormalities months or even years after the infection, some damage may be irreversible. Moreover, some fear that a COVID-19 infection may lead to a predisposition to develop other neurological disorders. For instance, some studies have suggested that COVID-19 patients may be more susceptible to accelerated aging (Tayeri et al., 2022) and to developing Alzheimer's Disease (Li et al., 2022, Zhou et al., 2021) or Parkinson's Disease (Albornoz et al., 2022).

### 4.4 Considerations for future COVID-19 neuroimaging studies

The first studies reporting cerebral abnormalities in larger study samples were observational, retrospective, included only hospitalized patients (admitted to the general ward or ICU), concerned a single imaging time point during the (sub)acute stage of the infection, and did not include a control group (neither a healthy nor another patient sample). As the pandemic continued, it became apparent that COVID-19-related symptoms persisted even after the infection – now labelled as the post-COVID-19 condition or long-COVID syndrome (World Health Organization (WHO), 2023) – and also affected outpatients. Consequently, more prospective studies have been designed to include both hospitalized and non-hospitalized patients, to evaluate long-term changes in the brain after a SARS-CoV-2 infection, to evaluate cerebral changes over time (i.e., multiple imaging time points), and to compare imaging findings to a control group.

Importantly, with these changes in study designs, it should be clearly reported whether studied samples included hospitalized (i.e., ICU or hospital ward) and/or non-hospitalized (i.e., outpatients) patients, as an indicator of the respiratory symptom severity during the acute stage of the infection (Table 1). Similarly, studies later in the pandemic often excluded patients with pre-existing neurological and/or neuropsychiatric disorders, whereas some studies early in the pandemic focusing on hospitalized patients did not set such exclusion criteria to avoid patient selection bias. Neurological history should be clearly reported considering its impact on the interpretation of neuroimaging findings in relation to COVID-19. Moreover, with the progression of the COVID-19 outbreak, distinct pandemic periods were dominated by different variants of the SARS-CoV-2 virus. Reporting during which period patients in the studied sample were infected is encouraged, since early virus variants (i.e., Alpha and Delta) were more likely to cause more severe respiratory or inflammatory symptoms than later variants (i.e., variants of the Omicron lineage) (Florensa et al., 2022, Hyams et al., 2023). The severity of the long-term cerebral damage reported may therefore be affected by the COVID-19 period in which patients were infected. It remains a challenge to (retrospectively) determine which SARS-CoV-2 variant caused the infection for each patient, but reporting the period of infection allows the inference of a probable dominant virus variant depending on the region.

In addition, later studies were not limited to cerebral abnormalities alone but also studied cerebral abnormalities in relation to other variables such as clinical characteristics, laboratory data, and cognitive performance. It has become evident that many COVID-19 patients still experience COVID-19-related symptoms well beyond supposed recovery of the initial infection (Davis et al., 2023). This long-COVID syndrome is characterized by several symptoms, such as fatigue and cognitive impairment. In this review, 81 % of the included advanced neuroimaging articles recorded subjective and/or objective residual symptoms at the time of imaging, of which 71 % reported a correlation between such symptoms and abnormal MRI findings. This suggests that advanced neuroimaging is a useful tool to identify neural substrates of long-COVID. However, to this date, no clear pattern of cerebral abnormalities has been identified to explain the complexity of long-COVID and reported results are inconclusive. For instance, Ajčević et al., (Ajčević et al., 2023) and Greene et al., (Greene et al., 2024) did not find a correlation between MRI abnormalities (perfusion and BBB leakage, respectively) and objective cognition (using the Montreal Cognitive Assessment; MoCA). However, these pMRI abnormalities were associated with subjective complaints (cognitive performance and brain fog). Moreover, where some articles included a cognition screening tool (such as the MoCA), others assessed individual cognitive domains. Notably, MRI abnormalities were most often associated with impaired memory and executive function, which could also possibly explain the frequent complaints of (mental) fatigue and brain fog in long-COVID.

To understand how the brain is affected by COVID-19 and isolate COVID-19-specific pathology from other causes, it is essential to review cerebral abnormalities in COVID-19 patients alone or in comparison to healthy individuals and to compare data from COVID-19 patients to other patient groups, such as patients with general critical illness or other neurological disorders. Additionally, as many outpatients also experience long-COVID symptoms (Desgranges et al., 2022), study samples should not be limited to (previously) hospitalized COVID-19 patients. Moreover, healthy controls should be carefully considered in the future, as many individuals have contracted the SARS-CoV-2 virus at some point and/or have been vaccinated. Another valuable way to address COVID-19-specific neuropathology is to compare pre- and post-infection imaging data within patients (Douaud et al., 2022, Zhang et al., 2023, Invernizzi et al., 2023, Debs et al., 2023). Although such datasets are sparse and previous imaging data may not have been collected with similar imaging protocols, biobanks and (regional) longitudinal cohort studies may offer opportunities. To date, the number of longitudinal designs is limited. However, follow-up data on patients can provide information on the possible transient nature of certain cerebral abnormalities. Above all, longitudinal data will become increasingly important to comprehend the long-term effects of a COVID-19 infection on the brain, in light of long-COVID symptoms, but also to study potential predisposition to neurological decline later in life. Importantly, with an increasing demand for longitudinal and long-COVID studies, researchers should opt for clearly reporting their definitions. Currently, using the term 'post-COVID' to refer to long-COVID syndrome may be confused with measurements *after* the infection. Lastly, multiparametric imaging protocols can provide evidence from different perspectives simultaneously, which is essential to capture the variety of pathological factors in COVID-19. Future studies should carefully take these considerations into account.

### 4.5 Future outlook on advanced neuroimaging in COVID-19

The multifaceted COVID-19 pathology demands a multiparametric imaging protocol. Inflammatory processes and mechanisms affecting the neurovascular unit play a significant role in COVID-19 neuropathology. Assessment of the functionality of the vessels demands further attention and could provide additional explanations for the observed perfusion abnormalities. Other parameters include CVR/CA status and pulsatility of smaller vessels, which can be assessed using MRI (Chen and Gauthier, 2021, van den Kerkhof et al., 2023). Another underexplored aspect of the neurovascular unit, which also plays a role in neuroinflammation, is the BBB. Multiple neuroimaging techniques can be used to visualize and quantify BBB impairment (Elschot et al., 2021, Moyaert et al., 2023). Some studies have suggested involvement of the BBB in COVID-19, but in vivo imaging of BBB impairment is sparse, with a single study in our search results assessing and providing limited evidence for BBB impairment in patients with long-COVID (Campbell et al., 2022). BBB impairment may explain long-COVID symptoms as it has previously been associated with cognitive impairment in patients with vascular mild cognitive impairment (Li et al., 2021). Moreover, white matter damage has been abundantly reported in COVID-19, with DTI allowing quantification of a range of measures, including anisotropy, fiber density, and volume of white matter tracts. DTI measures suggest demyelination in COVID-19, but this could be studied in more detail by quantifying the myelin content of white matter tracts using MRI or PET (Drenthen et al., 2020, van der Weijden et al., 2023). Abnormal myelination has been associated with multiple neurological disorders – including multiple sclerosis, Alzheimer's disease, and epilepsy (Drenthen et al., 2020) – and contributes to impaired cognitive processing (Abel et al., 2020), which is particularly useful to increase the understanding of long-COVID syndrome. Considering persisting inflammation and its link to neurodegenerative diseases, the cerebral waste clearance system has been postulated to play a role in long-COVID syndrome (Wostyn, 2021) but has received little attention. Impairment of this clearance system is thought to be significantly involved in neurodegenerative diseases (Nedergaard and Goldman, 2020), and has been associated with neuroinflammation and BBB impairment (Mogensen et al., 2021). Over the last decade, rapid developments have resulted in multiple ways to study the clearance system using MRI (van der Thiel et al., 2023). Lastly, the susceptibility of advanced neuroimaging techniques to more subtle changes has the potential to improve further by incorporating ultra-high field MRI (≥7 T). Indeed, ultra-high field MRI shows enhanced sensitivity to detect enlarged perivascular spaces (Langan et al., 2022) and punctuate lesions (Martin et al., 2022). However, the number of studies utilizing ultra-high field MRI is limited, leaving a window of opportunity for future imaging research.

## 5 Conclusion

A broad spectrum of cerebral abnormalities spread throughout the brain has been identified in COVID-19 using different advanced neuroimaging techniques. These findings are likely related to hypoxic, vascular, and inflammatory pathological mechanisms, as opposed to direct viral invasion of the brain in COVID-19. This is supported by the absence of viral protein in postmortem brain tissue. Advanced neuroimaging techniques facilitate a meticulous and dynamic outlook on these mechanisms, contributing to our understanding of cerebral pathology in COVID-19. Future studies should opt for advanced and multiparametric imaging techniques to fully comprehend how the brain is affected by COVID-19, especially in the long-term.

## CRediT authorship contribution statement

**Noa van der Knaap:** Writing – review & editing, Writing – original draft, Visualization, Validation, Methodology, Investigation, Conceptualization. **Marcel J.H. Ariës:** Writing – review & editing, Validation, Supervision, Methodology. **Iwan C.C. van der Horst:** Writing – review & editing, Supervision. **Jacobus F.A. Jansen:** Writing – review & editing, Validation, Supervision, Methodology, Conceptualization.

## Declaration of competing interest

The authors declare that they have no known competing financial interests or personal relationships that could have appeared to influence the work reported in this paper.

## Data Availability

No data was used for the research described in the article.
