# Supplementary material for: On the merits and potential of advanced neuroimaging techniques in COVID-19: A scoping review
Source: Neuroimage Clin. 2024 Mar 6;42:103589. doi: 10.1016/j.nicl.2024.103589 (PMC10938171; doi:10.1016/j.nicl.2024.103589)
Supplement: Supplementary data 1 [file mmc1.docx]

**On the merits and potential of advanced neuroimaging techniques in COVID-19: a scoping review**

Noa van der Knaap^1,2,3^, Marcel J.H. Ariës^1,3^, Iwan C.C. van der Horst^1,4^, Jacobus F.A. Jansen^2,3,5*^

^1^Department of Intensive Care Medicine, Maastricht University Medical Center, Maastricht, The Netherlands

^2^Department of Radiology & Nuclear Medicine, Maastricht University Medical Center, Maastricht, The Netherlands

^3^Research Institute of Mental Health & Neuroscience, Maastricht University, Maastricht, The Netherlands

^4^Cardiovascular Research Institute Maastricht (CARIM), Maastricht University, Maastricht, The Netherlands

^5^Department of Electrical Engineering, Eindhoven University of Technology, Eindhoven, The Netherlands

**Supplementary Table 1. Formulae for database searching.**

| **PUBMED** | **bioXriv + medXriv** |
| --- | --- |
| ((COVID-19[mh]) OR (SARS-CoV-2[mh]) OR (Coronavirus[mh])) AND ((Brain[mh]) OR (cerebral)) AND ((Magnetic Resonance Imaging[mh]) OR (computed tomography[mh]) OR (positron emission tomography[mh]) OR (Tomography, Emission-Computed, Single-Photon[mh]) OR (perfusion imaging[mh]) OR (DCE-MRI) OR (ASL) OR (DSC-MRI) OR (fMRI) OR (DTI) OR (DWI) OR (postmortem) OR (Autopsy[mh])) NOT ((cardiac) OR (pedriatic) OR (chest) OR (Case Reports[Publication Type]) OR (Review[Filter]) OR (Animal[Filter])) | (COVID-19 OR SARS-CoV-2 OR Coronavirus) AND (Brain OR cerebral) AND (MRI OR CT OR PET OR SPECT OR DCE-MRI OR ASL OR DSC-MRI OR fMRI OR DTI OR DWI OR postmortem OR Autopsy) |
